# Cryo-electron Tomography Reveals the Roles of FliY in *Helicobacter pylori* Flagellar Motor Assembly

**DOI:** 10.1128/msphere.00944-21

**Published:** 2022-02-02

**Authors:** Ping Lu, Huawei Zhang, Yuanzhu Gao, Xudong Jia, Zhe Liu, Daping Wang, Shannon Wing Ngor Au, Qinfen Zhang

**Affiliations:** a State Key Lab for Biocontrol, School of Life Sciences, Sun Yat-sen University, Guangzhou, China; b Department of Biomedical Engineering, Southern University of Science and Technology, Shenzhen, China; c School of Life Sciences, Faculty of Science, The Chinese University of Hong Kong, Shatin, Hong Kong, China; d Cryo-EM Facility Center, Southern University of Science and Technology, Shenzhen, China; e Guangdong Provincial Center for Disease Control and Prevention, Guangdong Provincial Institute of Public Health, Guangzhou, China; f Department of Orthopedics, Shenzhen Intelligent Orthopedics and Biomedical Innovation Platform, and Guangdong Artificial Intelligence Biomedical Innovation Platform, Shenzhen Second People’s Hospital, The First Affiliated Hospital of Shenzhen University Health Science Center, Shenzhen, China; University of Maryland Medical Center

**Keywords:** *Helicobacter pylori*, FliY, cryo-electron tomography, motor assembly, motility

## Abstract

Helicobacter pylori plays a causative role in gastric diseases. The pathogenicity of H. pylori depends on its ability to colonize the stomach guided by motility. FliY is a unique flagellar motor switch component coexisting with the classical FliG, FliM, and FliN switch proteins in some bacteria and has been shown to be essential for flagellation. However, the functional importance of FliY in H. pylori flagellar motor assembly is not well understood. Here, we applied cryo-electron tomography and subtomogram averaging to analyze the *in situ* structures of flagellar motors from wild-type strain, *fliY*-null mutant and complementation mutants expressing the N-terminal or C-terminal domain of FliY. Loss of full-length FliY or its C-terminal domain interrupted the formation of an intact C ring and soluble export apparatus, as well as the hook and flagellar filaments. Complementation with FliY C-terminal domain restored all these missing components of flagellar motor. Taken together, these results provide structural insights into the roles of FliY, especially its C-terminal domain in flagellar motor assembly in H. pylori.

**IMPORTANCE** Helicobacter pylori is the major risk factor related with gastric diseases. Flagellar motor is one of the most important virulence factors in H. pylori. However, the assembly mechanism of H. pylori flagellar motor is not fully understood yet. Previous report mainly described the overall structures of flagellum but had not focused on its specific components. Here, we focus on H. pylori flagellar C-ring protein FliY. We directly visualize the flagellar structures of H. pylori wild-type and FliY N-/C-terminal complementary strains by cryo-electron tomography and subtomogram averaging. Our results show that deletion of FliY or its C-terminal domain causes the loss of C ring, whereas deletion of FliY N-terminal does not affect C-ring assembly and flagellar structures. Our results provide direct evidence that C-ring protein FliY, especially its C-terminal domain, plays an indispensable role in H. pylori motor assembly and flagellar formation. This study will deepen our understanding about H. pylori pathogenesis.

## INTRODUCTION

Flagellum-mediated movement is a well-evolved motility system in prokaryotes and a key virulence factor for many pathogenic bacteria to colonize and survive in the host (1). Formation of a functional bacterial flagellum relies on the self-assembly of supramolecular complex, which can be divided into three parts: the filament, the hook, and the basal body. The filament is made up by polymerization of thousands of flagellin molecules into a long helical propeller-like structure. The short axial structure of hook functions as a flexible joint that connects the filament and the basal body. The core structure of basal body is a nano-bidirectional rotary device that consists of transmembrane stator complex and stacks of ring structure (the MS ring in the inner membrane and the C ring in the cytoplasm) (2). Rotation of flagellar filament requires ion motive force through the stator and subsequent molecular interaction between the stator and the C ring for torque generation (3).

The basal body, in particular the C ring, is a highly dynamic structure and is critical for motor function and flagellar synthesis. In model microorganisms such as Escherichia coli and Salmonella Typhimurium, the C ring is composed of well-conserved switch proteins, including ∼26 copies of FliG, ∼34 copies of FliM, and ∼100 copies of FliN (4–6). These three switch proteins are indispensable for flagellar assembly, and *fliG*-, *fliM*-, or *fliN*-null mutant strains results in nonflagellation phenotype (7–9). FliG located at the upper C ring is involved in the torque generation and transmission, through the interactions of its C-terminal domain with the cytoplasmic domain of MotA of the stator and of its N-terminal domain with the cytoplasmic domain of FliF at the MS-C ring junction, respectively (10–14). The middle of the C ring is formed by FliM, which binds to the middle domain of FliG and coordinates chemotactic response. Rotational switching between clockwise and counterclockwise is induced by the binding of response regulator CheY to FliM (15, 16). The lower C ring is a drum-shaped structure formed by FliM-FliN_4_ (6, 17–19). Export of flagellar proteins involves docking of the soluble ATPase complex through FliN to the C ring and attachment of the transmembrane type III export apparatus within the MS ring (19, 20).

While the overall structure of flagellar motor is evolutionary conserved across bacterial species, variations in the components and structural architectures of flagellar motors have been found. For the C ring, Bacillus subtilis possess FliY, a chimera composed of CheC/FliM-like domain and a FliN-like domain, as a substitution of FliN (21). Complementation of FliY can restore motility but not chemotaxis in Salmonella Typhimurium Δ*fliN* mutant strain, indicating distinct roles of FliY in flagellar regulation (21). In *Epsilonproteobacteria* Helicobacter pylori, the C ring possesses both FliN and FliY. Although H. pylori FliY and B. subtilis FliY resemble each other in terms of domain organization, the N-terminal end of FliY in B. subtilis has a CheY-binding motif (22). Interestingly, recent studies in Campylobacter jejuni, a species closely related to H. pylori, reveal that C. jejuni motor C ring is also involved in cell division. This diverse function may relate to the coexistence of FliY and FliN (23). Our group has previously determined the crystal structure of FliY-FliN complex composing of their C-terminal domains and demonstrated the binary complex is required for the association with FliH (22). However, *in situ* structural evidence for a better understanding of the structural and functional significance of FliY in the H. pylori flagellar motor is lacking.

Here, we applied cryo-electron tomography (cryo-ET) and subtomogram averaging methods to visualize the *in situ* structures of flagellar motors from H. pylori wild-type (WT) and Δ*fliY*, FliY_N_, and FliY_C_ mutant strains. Our results suggest that H. pylori FliY participates in the C-ring formation and also soluble export apparatus assembly, mainly through its C-terminal domain.

## RESULTS

### Architecture of the intact flagellar motor in *H. pylori* wild-type strain.

**FIG 1:**
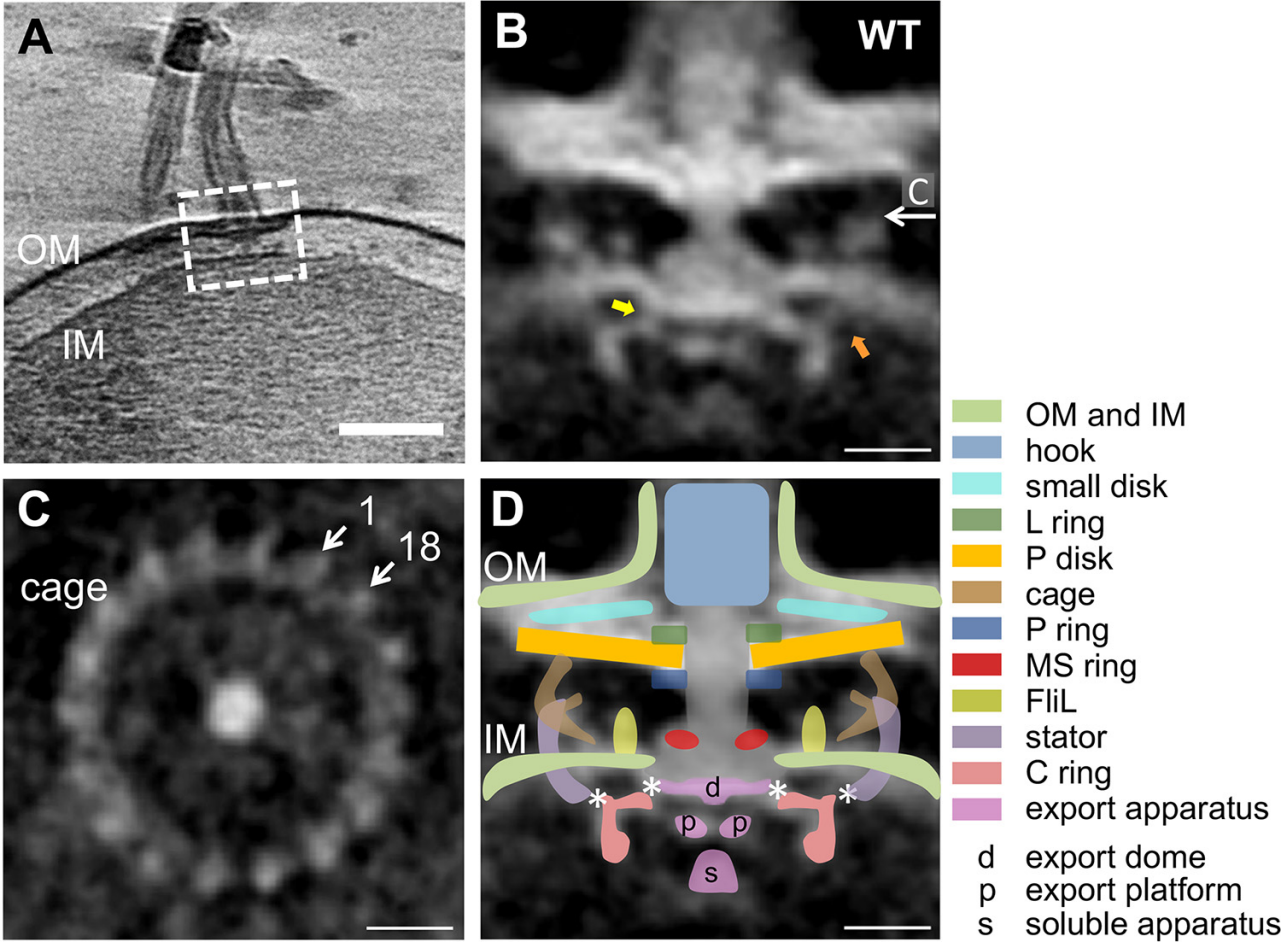
Flagellar motor structure of H. pylori wild-type strain. (A) Slice from a tomogram of H. pylori wild-type strain shows the flagellar motor’s location (white dashed line box) and the cell envelope, including the outer membrane (OM) and inner membrane (IM). (B) Slice from the averaged map of motor without any symmetry imposed. The arrows indicate the connection between the C ring and the stator (right arrow in orange), the C ring, and MS ring (left arrow in yellow). (C) A transversal slice through the averaged motor shows the striking 18-fold symmetry at the height indicated by the white arrow in panel B. (D) Scheme diagram of the flagellar motor with different parts superimposed on panel B. The putative FliG-MS ring contact site and FliG ring-stator contact site are marked by asterisks. Color schemes for different parts are indicated. Scale bar, 100 nm (for panel A) and 20 nm (for other panels).

Among 24 tilt series of tomograms from H. pylori G27 wild-type (WT) strain, a total of 59 subvolumes of motors were selected for the calculation of the final averaged map. The *in situ* structure of the averaged motor was determined at a resolution of 6.5 nm (Fig. 1A and B; see also Fig. S1 and Table S1 in the supplemental material).

10.1128/msphere.00944-21.1 FIG S1 The Fourier shell correlation (FSC) curves for the final cryo-ET maps without symmetry imposition. The resolutions for maps from the WT, *ΔfliY*, and FliY_C_ strains were estimated to be 6.5, 7.3, and 5.5 nm, respectively, based on an FSC 0.143 criterion. Download FIG S1, TIF file, 1.0 MB. Copyright © 2022 Lu et al. 2022 Lu et al. https://creativecommons.org/licenses/by/4.0/ This content is distributed under the terms of the Creative Commons Attribution 4.0 International license.

10.1128/msphere.00944-21.3 TABLE S1 Summary of flagellar motors in different H. pylori strains. Download Table S1, DOCX file, 0.01 MB. Copyright © 2022 Lu et al. 2022 Lu et al. https://creativecommons.org/licenses/by/4.0/ This content is distributed under the terms of the Creative Commons Attribution 4.0 International license.

A striking 18-fold symmetry feature is visible from the cross-section around the periplasmic cage-like region in the map before imposing any symmetry (Fig. 1C). Symmetry features in other parts of the motor were difficult to determine.

Cryo-ET structures of flagellar motor from different bacterial species, including E. coli, Borrelia burgdorferi, C. jejuni, and H. pylori, have been reported (18, 24–26). With reference to these motor structures, different substructures were annotated to our map for better visualization (Fig. 1D). Several structural elements, including the L ring, the P ring, the rod, the MS ring, the C ring, the stator, and the export apparatus, can be easily identified in the resolved motor structure. Similar to the previous study (26), specific components, such as the cage, the P disk, and a smaller disk above the P disk, as well as the putative FliL ring, can also be detected in our resolved map (Fig. 1B and D).

The upper part of the motor is featured with various disk structures. Around the rod, the L/P ring connect to the P disk, as shown in previous research (26). Another smaller disk between P disk and the outer membrane is also observed here (Fig. 1B and D). Following the P disk is the arch-shape cage-like periplasmic structure with 18-fold symmetry and a diameter range from ∼80 to ∼85 nm from the upper to the middle position (Fig. 1B and D). Near the cage, there is a ring structure whose diameter is close to that of the cage and appears to anchor to the inner membrane. Based on its location and shape, together with references to the previously reported structures and molecular biological evidences (25–27), we annotate this structure as the stator. There is also evident density between the cage/stator and the MS ring, which is apparently embedded in the inner cytoplasmic membrane and extended to the periplasm. This structure should be the previous reported FliL (26, 28, 29).

Inside and connecting to the inner membrane is the C ring, with a diameter of ∼55 nm. The averaged map reveals that there is density extending from the peripheral of upper C ring to the stator studs. In addition, density connecting MS ring and the inner edge of C ring is visible (Fig. 1B and D). The export apparatus with a diameter of about 11 nm is visible inside the C ring and connect to the cytoplasmic side of the MS ring. The intact density of export apparatus can further be identified to be three parts: export dome, platform, and soluble apparatus (Fig. 1B and D).

### FliY plays critical roles in the assembly of C ring and export apparatus.

**FIG 2:**
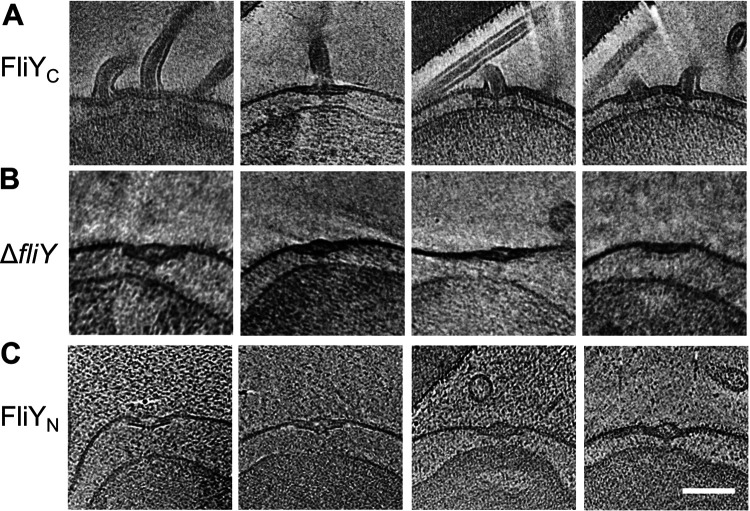
Gallery of motors from Δ*fliY*, FliY_C_, and FliY_N_ mutant strains. Axial slices of the tomograms from FliY_C_, Δ*fliY*, and FliY_N_ mutant strains were extracted to show the morphology of the motors. Representative images are shown for FliY_C_ (A), Δ*fliY* (B), and FliY_N_ (C) strains. One or more motors can be found for the FliY_C_ strain, and the distances between the inner and outer membranes are similar between the pole areas and other areas. For Δ*fliY* and FliY_N_ strains, no obvious whole motors can be found, and a mass of inner membrane are detached from the outer membrane. About 82% images of the *ΔfliY* strain and 80% of the images of FliY_N_ strains are observed with membrane deformation, as shown in panels B and C. Scale bar, 100 nm (all panels are in the same scale).

To investigate the importance of FliY in motor assembly, we further analyzed the *in situ* structures of flagellar motors from several mutant strains, including the Δ*fliY* mutant and the complemented strains FliY_N_ (expressing FliY residues 1 to 205) and FliY_C_ (expressing FliY residues 206 to 287) (Fig. 2). The flagellation and membrane integrity in FliY_C_ strain appear to be similar to that of the wild-type strain (Fig. 2A). In line with previous phenotypic studies (7, 30), the cryo-EM images of the Δ*fliY* and FliY_N_ strains revealed that none of the cells has flagellar filament. In addition, deformation of the cell membranes was observed in the *ΔfliY* strain (∼82% images) and the FliY_N_ strain (∼80% images), as shown in Fig. 2B and C.

### Structure of flagellar motor in the *H. pylori* Δ*fliY* strain.

**FIG 3:**
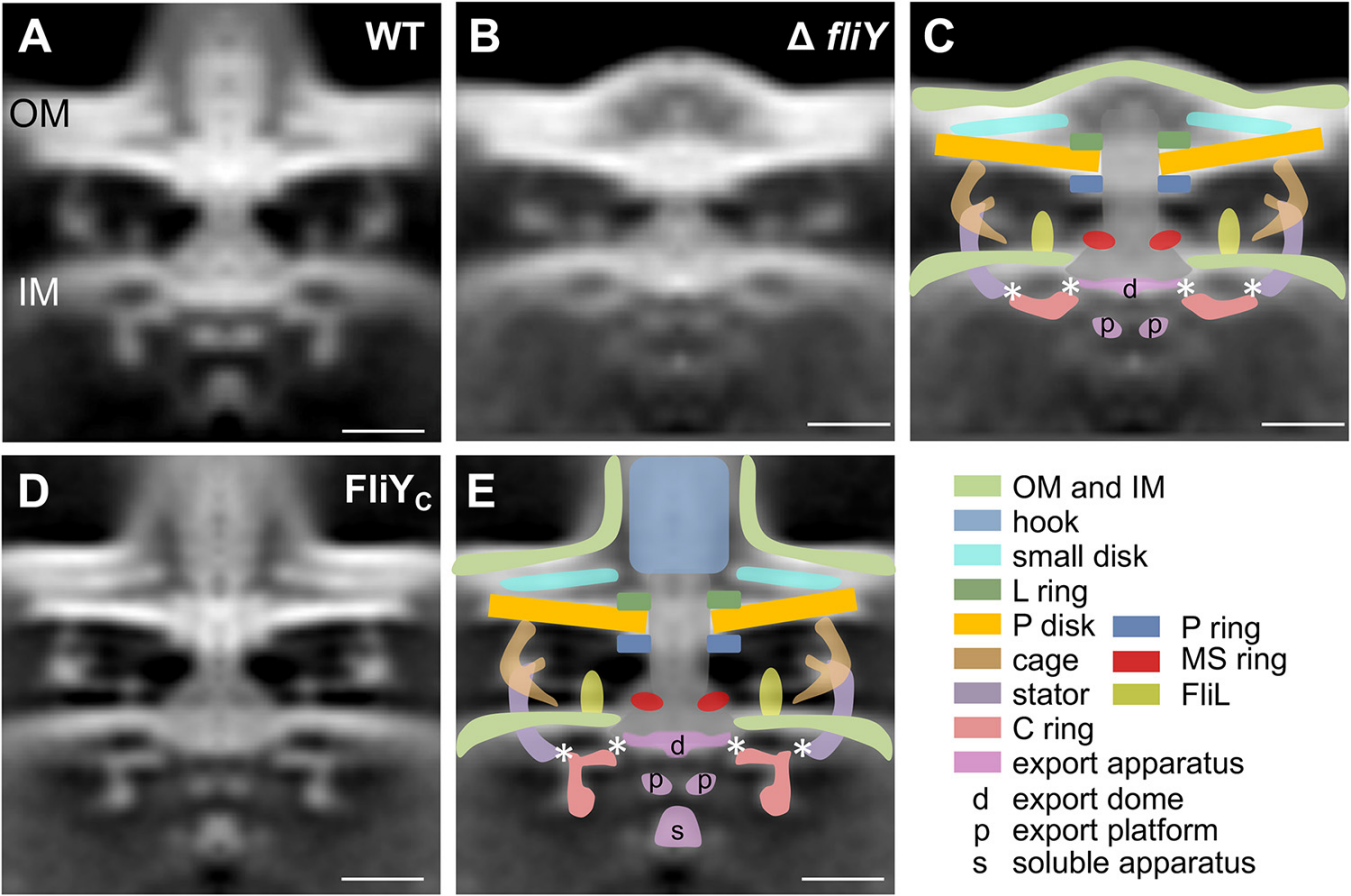
Flagellar motor structures of H. pylori Δ*fliY* and FliY_C_ strains. (A) The motor structure from WT strain in Fig. 1B was imposed with C_18_ symmetry. (B) Axial slice of the averaged motor map imposed with C_18_ symmetry in the Δ*fliY* strain. (C) Scheme diagram of different flagellar parts superimposed on panel B. (D) A slice from the tomogram of the H. pylori FliY_C_ strain shows normal flagellar formation in the FliY_C_ strain. (E) Scheme diagram of different flagellar parts superimposed on panel D. The putative FliG-MS ring contact site and FliG ring-stator contact site are marked by asterisks. The color schemes in panels C and E are the same as that of Fig. 1D. Scale bar, 20 nm.

The molecular architecture of the flagellar motor in the Δ*fliY* strain was determined by cryo-ET and subtomogram averaging. Since no flagella were detected, one apical region of each cell was randomly selected for imaging. A series of 76 tilt tomograms was collected, and only <40% tomograms contained the motor structures. A total of 28 motor subtomograms were extracted from all of the tomograms, and 13 motor subtomograms were finally selected for further subtomogram averaging (see Table S1 and Fig. S1). To improve the signal-to-noise ratio and to compare the structures of WT and other mutant strains, the final maps of the flagellar motor from WT (Fig. 1B) and the Δ*fliY* strains were imposed with C_18_ symmetry, as determined in the WT structure (Fig. 3).

Compared to the motor structure of the WT strain, dramatic structural differences were found in the Δ*fliY* mutant strain (Fig. 3A to C). There are no obvious densities corresponding to the middle-lower parts of the C ring and to the soluble export apparatus in the Δ*fliY* mutant strain. Furthermore, the densities for the hook and the filament are also lost in the Δ*fliY* strain (Fig. 3A to C). However, the densities for the L/P ring, the P disk, together with its upper small disk, cage, stator, FliL ring, MS ring, the rod, the upper part of C ring, the export dome, and platform, can still be mapped with confidence (Fig. 3A to C). These results indicate that FliY is indispensable for the assembly of an intact C ring, in particular the middle-lower region where FliM and FliN localize.

For FliY_N_ mutant, 45 tilt series tomograms were collected but less than half of the tomograms could identify the motor (see Table S1). The result of the subtomogram average was similar to that of the Δ*fliY* strain (data not shown). The quality is unsatisfactory due to the small quantity of data and deformation of the cells (Fig. 2C). These results suggest that the C-terminal domain of FliY is critical for the motor synthesis and flagellation in H. pylori.

### Structures of flagellar motors in *H. pylori* FliY_C_ strain.

A previous study showed that the C-terminal of FliY forms a heterodimer with FliM and FliN *in vitro* and that the two binary complexes are associated with the flagellar regulatory protein FliH (22). Here, the cryo-ET structure of flagellar motor from the FliY_C_ mutant provides direct structural evidence to illustrate the functional importance of the C-terminal of FliY in the motor assembly. After collecting 33 tomograms from the FliY_C_ mutant, a total of 142 subtomograms were averaged to obtain the final map of the motor (see Table S1 and Fig. S1). The final map of the motor structure in the FliY_C_ strain was then imposed with C_18_ symmetry (Fig. 3D and E) to compare it with that of the WT and Δ*fliY* strains.

Compared to the motor structure in Δ*fliY* strain, those missing densities, including the C ring and soluble export apparatus, as well as the hook and filaments in Δ*fliY* mutant, are all restored (Fig. 3). This result indicates that the N-terminal domain of FliY does not play a vital role in the assembly of C ring and export apparatus in H. pylori. Although the overall motor structure resembles that of the wild type, there are subtle differences in several components of the motor structure. For example, there is a fragile density links the cage-like structure to FliL, the length of the rod seems shorter, and the density for stator becomes weaker (Fig. 3D and E). However, due to the low resolution of the motor, it is hard to tell whether these differences are real and resulted from the deletion of N-terminal of FliY in H. pylori.

## DISCUSSION

It is popularly accepted that the stable FliM-FliN complex localizes at the lower part of C ring through binding to FliG in the model of motor switch complex (31). FliY is thought to be located at the bottom C ring due to its good fit with the crystal structure of FliY_M_ in the previously reported *Leptospira* flagellar motor structure (32, 33). However, extra densities corresponding to the putative FliY in the C ring were not found in the H. pylori motor structure solved at a higher resolution (26).

Here, comparative studies of the flagellar motors from different H. pylori strains provide direct *in situ* evidences for the roles of FliY in motor integrity. When FliY or the C-terminal domain of FliY was deleted, the density of the putative FliM-FliN complex is missing, while the upper parts of the C ring corresponding to the putative FliG ring still existed (Fig. 3B and C). Taken together with previous biochemical data showing the C-terminal FliY interact with the C-terminal FliM and C-terminal FliN in H. pylori (22), it is possible that the C-terminal FliY colocalizes in the middle-lower C ring, together with FliM. This also helps to explain why the deletion of FliY or its C-terminal will affect the C-ring assembly. Although FliY does not interact with FliG (22), these results indicate that FliY is involved in docking FliM-FliN to FliG.

The C ring is composed of FliG, FliM, and FliN for most bacteria, while FliY is an additional component in H. pylori. A previous report shows that FliN and the C terminus of FliY in H. pylori show similar domain organizations (22). Sequence alignment of FliY and FliN from Helicobacter pylori, Campylobacter jejuni, Escherichia coli, and Salmonella enterica indicates that the C-terminal end of FliY is evolutionarily conserved with FliN (see Fig. S2). The C-terminal end of FliY may serve as an alternative form of FliN to play a role in the motor assembly; thus, deletion of C-terminal of FliY may affect the motor integrity, as reported here.

10.1128/msphere.00944-21.2 FIG S2 Multiple sequence alignment of FliY and FliN from different bacterial species. Protein sequences of FliN and FliY were aligned using Clustal Omega (F. Madeira et al., 2019, Nucleic Acids Res 47:W636–W641, 10.1093/nar/gkz268). The N-terminal and C-terminal ends of FliY (based on H. pylori FliY sequence) are boxed in red and green, respectively. Identical residues in the C-terminal are indicated in boldface. H. pylori, Helicobacter pylori; C. jejuni, Campylobacter jejuni; E. coli, Escherichia coli; S. enterica, Salmonella enterica; *L. biflexa*, Leptospira biflexa. Download FIG S2, TIF file, 0.9 MB. Copyright © 2022 Lu et al. 2022 Lu et al. https://creativecommons.org/licenses/by/4.0/ This content is distributed under the terms of the Creative Commons Attribution 4.0 International license.

In our studies, the H. pylori wild-type strain and all FliY mutant strains share some common structures in the flagellar motor, mostly located in the outer membrane, the inner membrane, or the periplasmic space, including the P disk, small disk, L ring, P ring, MS ring, cage-like structure, and FliL (Fig. 3). However, it appears that the length of the rod from the *ΔfliY* strain is shorter than the one from the wild-type strain (Fig. 3A to C). This could be due to the deformation of the cell membrane of the mutant strain that induced some difficulty in alignment during motor structure construction. However, we cannot rule out the possibility that FliY may have multiple roles and that its deletion will have an impact beyond the C ring. Other than the changes in the rod, FliY may also affect the membrane integrity. As shown in Fig. 2B and C, the outer membrane and inner membrane are separated, and the whole membrane is deformed, indicating that FliY may indirectly affect the integrity of membrane structure, possibly through the export of some key regulator of membrane integrity.

One distinct feature of the H. pylori motor is the cage-like structure, which may play a role in torque generation in high-viscosity environments (26). In our studies, both *ΔfliY* and FliY_N_ strains harbor the cage-like structure, as observed in the wild-type strain (Fig. 3), indicating that FliY does not affect its formation, and likely the assembly of the cage-like structure occurs prior to that of the C ring.

The C-terminal domain of FliY has proved to be critical in flagellar formation, while the roles of N-terminal domain still remain unclear. In our studies, the motor structures of the *ΔfliY* and FliY_N_ strains look quite similar, indicating that the N-terminal domain is a dispensable part for flagellar assembly. In the future, high-resolution cryo-EM/cryo-ET maps of the flagellar motors in conjunction are needed to relate the evolution of this unique switch component to H. pylori adaptation.

## MATERIALS AND METHODS

### *H. pylori* culture.

The H. pylori wild-type strain G27, the Δ*fliY* strain, and the FliY_N_ and FliY_C_ complementation strains expressed with the N- or C-terminal domain of FliY, respectively (7, 30) were inoculated into 10 mL of brucella broth supplemented with 10% heat-inactivated fetal bovine serum. The bacterial culture was grown at 37°C under microaerophilic conditions for about 24 h until the optical density at 600 nm reached ∼2. Cells were harvested by centrifugation at 2,000 × *g* for 10 min, followed by washing with sterile phosphate-buffered saline (PBS). The cell pellet was resuspended in PBS and subjected to cryo-tomography studies.

### Sample preparation for cryo-ET.

Cells suspended in PBS were gently mixed with 15-nm colloidal gold (used as fiducial marks) just before plunge freezing. For the cryo-EM grid preparation, 3 μL of the sample was deposited onto a freshly glow-discharged R3.5/1, 300-mesh Quantifoil carbon-coated grid for 1 min. The grid was then blotted with filter paper and, after 5 s, this was followed by rapid plunge freezing in liquid ethane using a Vitrobot Mark IV (Thermo Scientific). The grids were stored in liquid nitrogen until data collection.

### Data collection of cryo-electron tomography.

Images of the H. pylori wild-type G27 strain and Δ*fliY* strain were acquired on TF20 electron microscope operating at 200 kV controlled by tomography (Thermo Scientific). Single-axis tilt series images in a range of −60° to 60° with 2° increments, at an ∼8-μm underfocus with a cumulative dose of ∼150 e^–^/Å^2^ and at a nominal magnification of 29,000× with a pixel size of 0.38 nm, were recorded on a 4,028 × 4,028-pixel Eagle CCD camera (Thermo Scientific).

Single-axis tilt series images of FliY_C_ and FliY_N_ strains were recorded at a range of −60° to 60° at 3° increments with cumulative dose of ∼120 e^–^/Å^2^ in a Titan Krios electron microscope equipped with a K3 direct detection device (Gatan) for FliY_C_ and with a K2 direct detection device (Gatan) for FliY_N_ operated at 300 kV. For FliY_C_, each tilt-angle movie with 12 frames was recorded at an ∼3-μm underfocus with a nominal magnification of 33,000×, yielding a pixel size of 0.2665 nm. For FliY_N_, each tilt-angle movie with 10 frames was recorded at an ∼3-μm underfocus with a nominal magnification of 53,000×, yielding a pixel size of 0.2668 nm.

### Tomogram reconstruction and subvolume averaging.

For the wild-type and Δ*fliY* tomographic data, IMOD was used for the CTF corrections and tomogram reconstructions with the data binned 4 with a final pixel size of 1.52 nm (34, 35). Subvolumes containing the flagellar motor were extracted from the tomograms and initially aligned through iterations of rotation and transition, as previously described (36, 37). The initial averaged map was then used as a reference for the next rounds of alignment until the results became stable. For the FliY_N_ and FliY_C_ tomographic data, raw movies were corrected for beam-induced motion using Warp (38). Tomograms were reconstructed by Dynamo (39) and IMOD (34) using the data binned to final pixel sizes of 1.066 nm for FliY_C_ and 0.8 nm for FliY_N_. The subvolumes were extracted and aligned using Dynamo and further refined using Relion (40).

C_18_ symmetry was imposed on the final maps to improve the signal-to-noise ratio. The resolution of final averaged subtomograms before imposing symmetry were calculated by comparing two halves of the data using Fourier shell correlation with a threshold of 0.143.

### Visualization of 3D reconstruction.

The surface rendering of the flagellar motors was carried using UCSF Chimera (41) with watershed segmentation (42).

### Data availability.

Maps of H. pylori motors from wild-type, Δ*fliY*, and FliY_C_ strains have been deposited in the Electron Microscopy Data Bank under accession numbers EMD-32012, EMD-32014, and EMD-32015, respectively.
