# Supplementary figures and images for: Cryo-electron Tomography Reveals the Roles of FliY in Helicobacter pylori Flagellar Motor Assembly
Source: mSphere. 2022 Feb 2;7(1):e00944-21. doi: 10.1128/msphere.00944-21 (PMC8809382; doi:10.1128/msphere.00944-21)

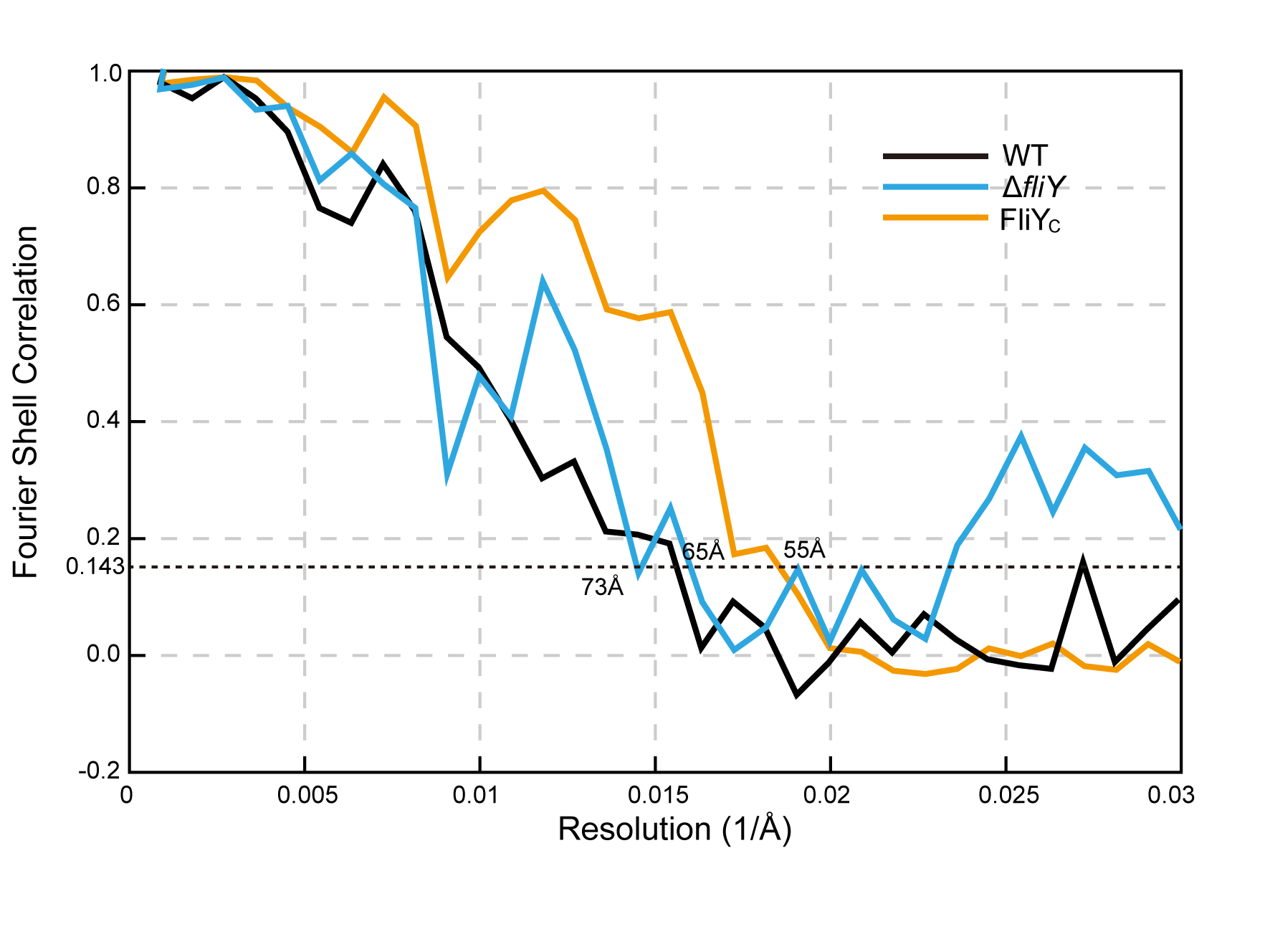

Supplement: FIG S1 [file msphere.00944-21-sf001.tif]

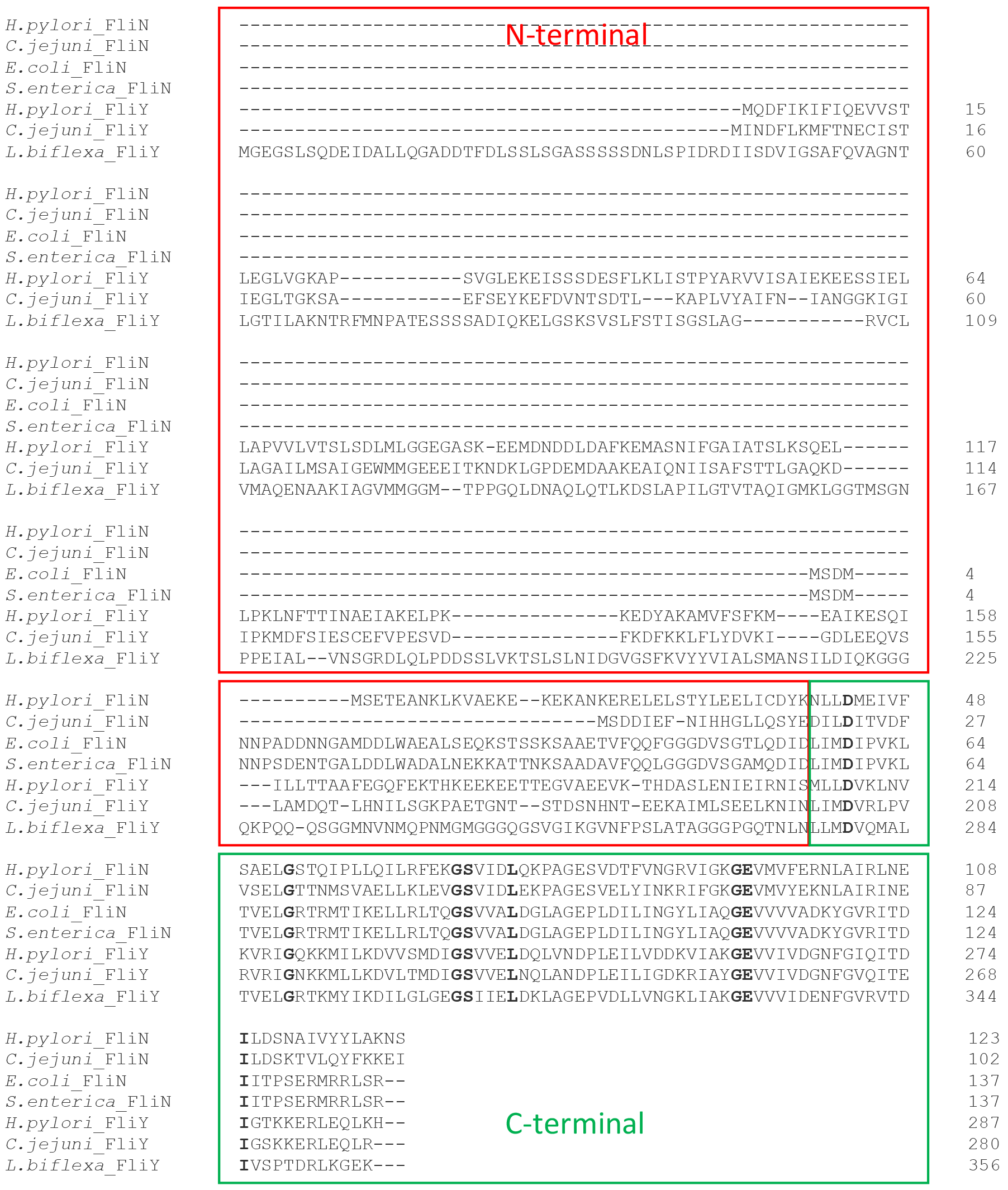

Supplement: FIG S2 [file msphere.00944-21-sf002.tif]
