# Supplementary material for: Cryo-electron Tomography Reveals the Roles of FliY in Helicobacter pylori Flagellar Motor Assembly
Source: mSphere. 2022 Feb 2;7(1):e00944-21. doi: 10.1128/msphere.00944-21 (PMC8809382; doi:10.1128/msphere.00944-21)
Supplement: TABLE S1 [file msphere.00944-21-st001.docx]

**Table S1． Summary of flagellar motors in different *H. pylori* strains**

| *H. P.* strain | nm/pixel  (reconstruction) | Defocus | Tilt-series acquired | Sub-tomo  (total) | Sub-tomo selected | Resolution  (nm) |
| --- | --- | --- | --- | --- | --- | --- |
| WT | 1.52 | ~8 μm | 24 | 61 | 59 | 6.5 |
| Δ*fliY* | 1.52 | ~8 μm | 76 | 28 | 13 | 7.3 |
| FliY_C_ | 1.07 | ~3 μm | 33 | 142 | 142 | 5.5 |
| FliY_N_ | 0.8 | ~3 μm | 45 | 32 | - | - |
